# Supplementary material for: Versatility in acyltransferase activity completes chicoric acid biosynthesis in purple coneflower
Source: Nat Commun. 2021 Mar 10;12:1563. doi: 10.1038/s41467-021-21853-6 (PMC7946891; doi:10.1038/s41467-021-21853-6)
Supplement: Supplementary file 1 — Supplementary Information [file 41467_2021_21853_MOESM1_ESM.pdf]

Versatility in acyltransferase activity completes chicoric acid biosynthesis in purple coneflower

Fu and Zhang *et al.*

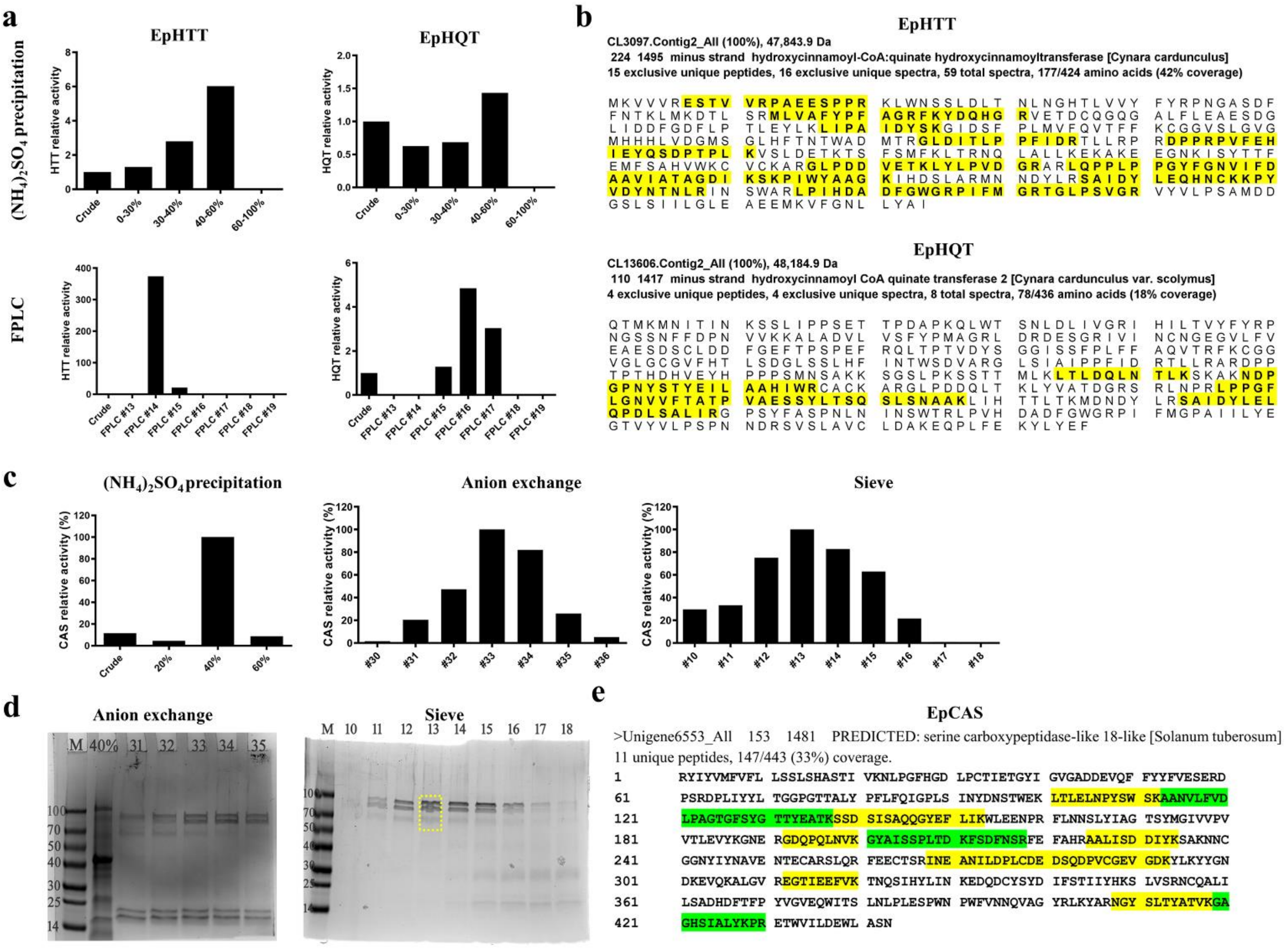

Supplementary Fig. 1. Purification and identification of acyltransferases. a, Relative EpHTT and EpHQT activities of fractions from $(NH_4)_2SO_4$ precipitation and FPLC separation. **b**, Identification of EpHTT and EpHQT using peptide mass mapping. The identified peptides are highlighted in yellow. **c**, Relative EpCAS activities of fractions from $(NH_4)_2SO_4$ precipitation and FPLC. **d**, SDS-PAGE analysis during separation of EpCAS. #33 with the highest activity from anion exchange was further separated using sieve. The bands used for peptide mass mapping are marked as red box. This experiment was repeated independently three times with similar results. The unit for molecular weight marker is kDa. **e**, Identification of EpCAS using peptide mass mapping. The identified peptides are highlighted in yellow and green. Source data are provided as a Source Data file.

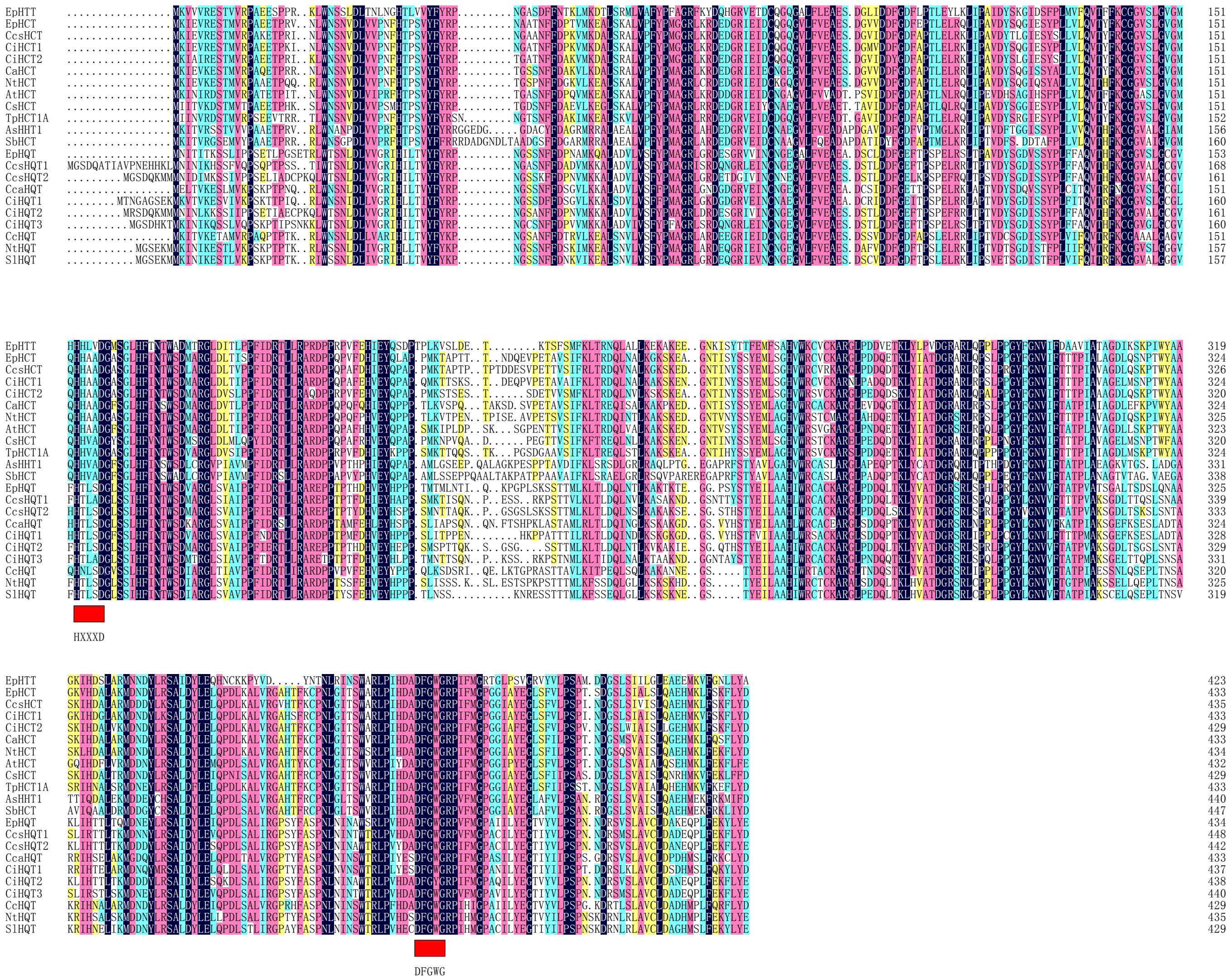

Supplementary Fig. 2. Multiple sequence alignment of EpBAHDs. Conserved HXXXD and GFGWG motifs are marked by red boxes. GeneBank ID of the proteins used: CcsHCT, AFL93686; CiHCT1, ANN12608; CiHCT2, ANN12609; NtHCT, CAD47830; CaHCT, CAJ40778; TpHCT1A, ACI16630; AtHCT, AED95744; SbHCT, 4KE4_A; AsHHT1, BAC78633; CcsHQT1, CAM84302; CcsHQT2, CAR92145; CcaHQT, ABK79690; CiHQT1, ANN12610; CiHQT2, ANN12611; CiHQT3, ANN12612; CcHQT, ABO77957; NtHQT, CAE46932; SlHQT, CAE46933; CsHCT, AEJ88365. The colors of dark blue, hot pink, cyan and light yellow represent 100%, ≥75%, ≥50% and ≥33% amino acid identity, respectively.

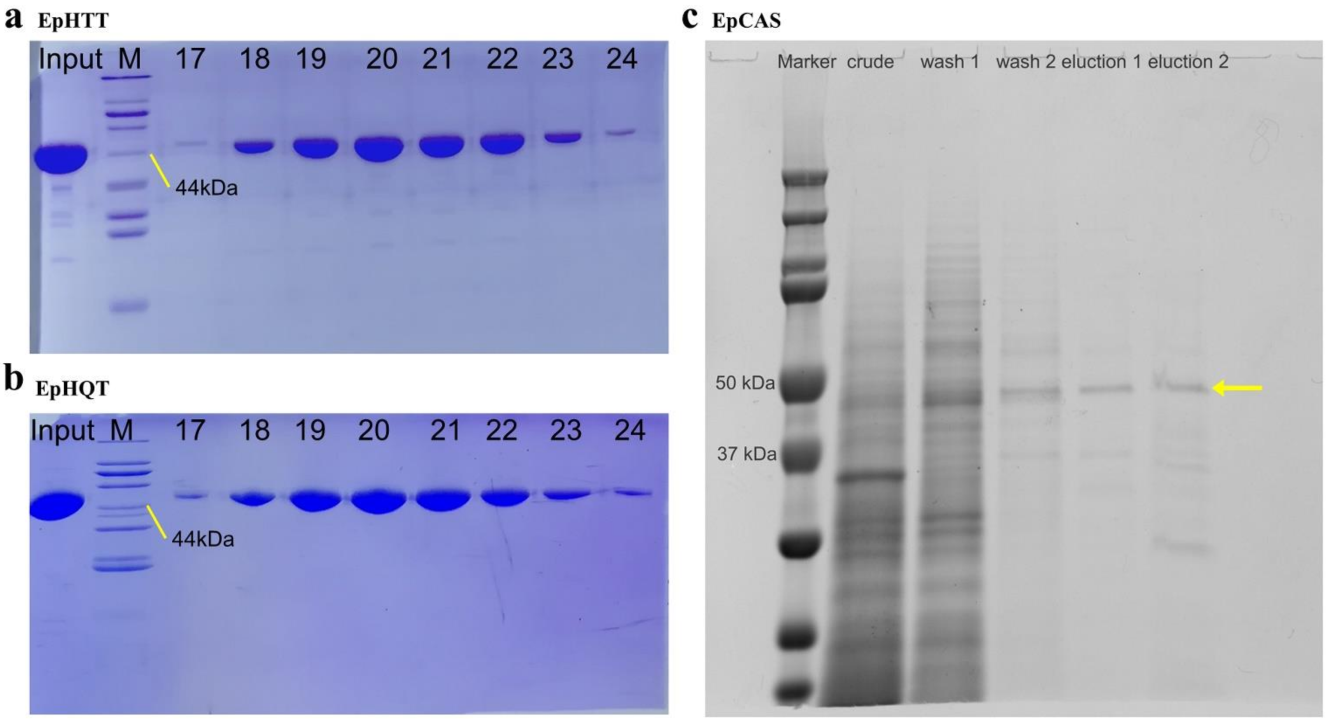

Supplementary Fig. 3. SDS-PAGE analysis of recombinant enzymes. a, EpHTT. **b**, EpHQT. **c**, EpCAS. Panels a and b were taken by a digital camera after staining with Coomassie blue. The track labelled Input presents the sample used for FPLC; M shows the M_r markers; 17-24 represent the fraction numbers collected following FPLC. Image shown as panel c was taken by a Bio-Rad ChemiDOC^{TM} Touch instrument in stain-free mode. Elution 1 was used for *in vitro* enzyme assays. These experiments were repeated three times with similar results. Source data are provided as a Source Data file.

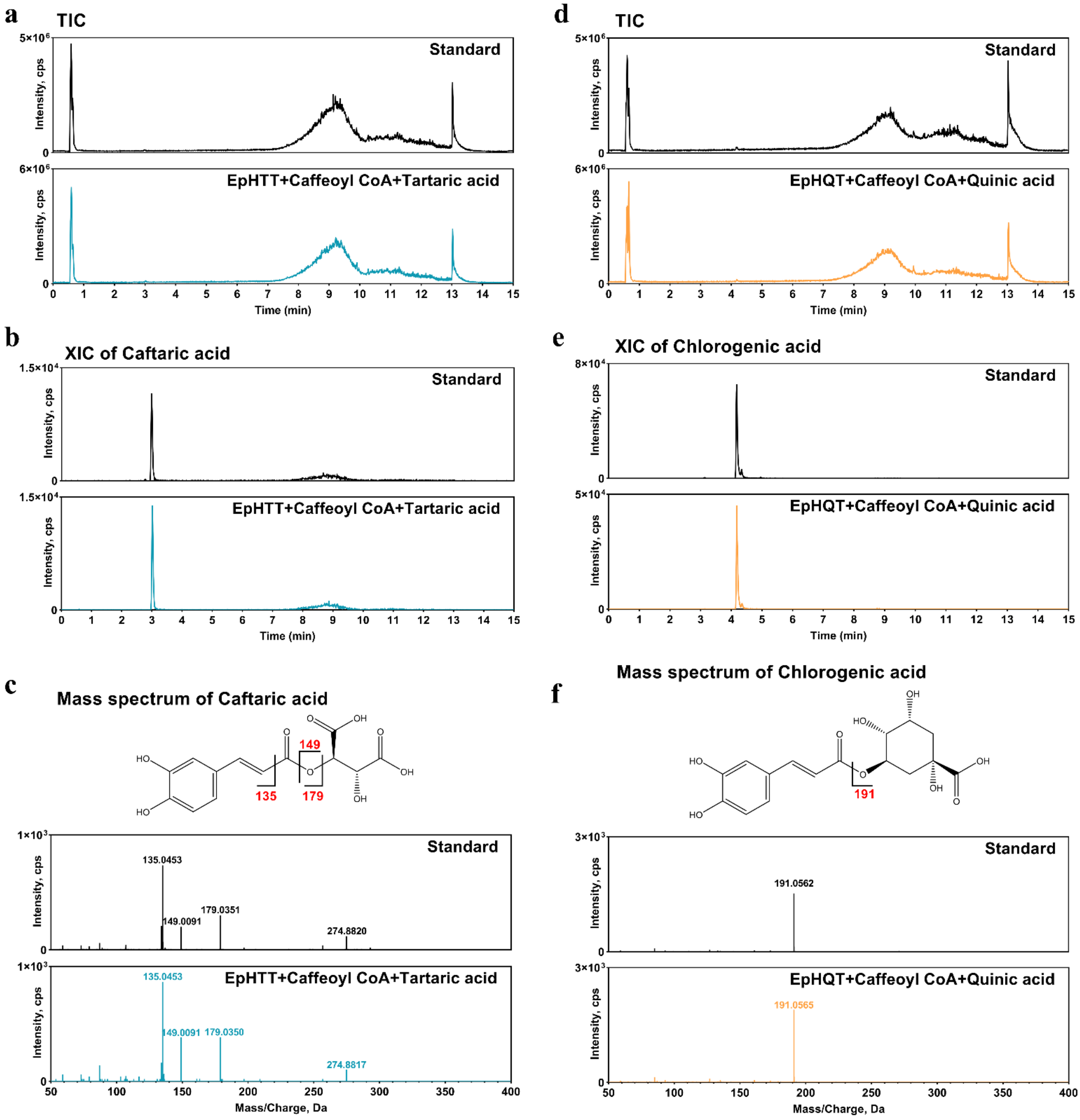

Supplementary Fig. 4. Identification of *in vitro* enzyme products of EpHTT and EpHQT using LC-HRMS. a, TIC of EpHTT reaction. **b**, XIC of caftaric acid. **c**, The mass spectra of the caftaric acid product and standard. **d**, TIC of EpHQT reaction. **e**, XIC of chlorogenic acid. **f**, The mass spectra of the chlorogenic acid product and standard.

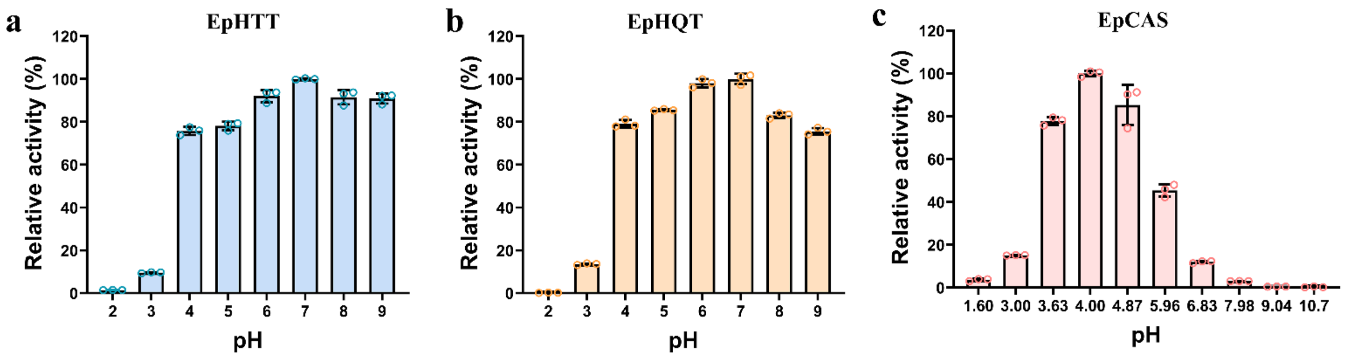

Supplementary Fig. 5. Acyltransferases activities at different pH values. a, EpHTT and **b**, EpHQT. Both BAHD acyltransferases showed highest activity at pH 7. **c**, EpCAS showed highest activity at pH 4. Data show mean±s.d. (n=3 independent experiments). Source data are provided as a Source Data file.

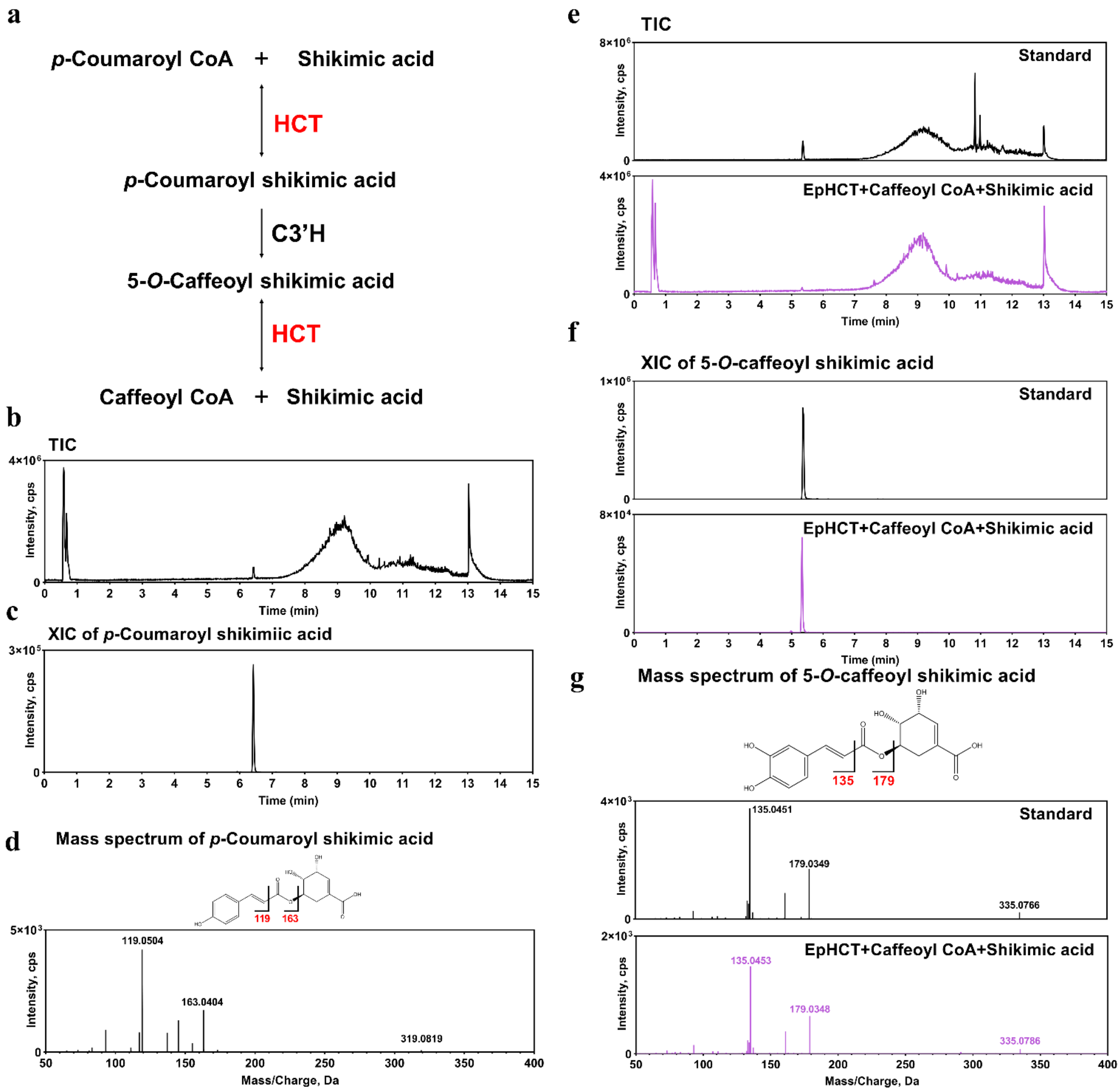

Supplementary Fig. 6. *In vitro* characterization of enzyme activity of EpHCT. a, Proposed reactions catalyzed by EpHCT. **b**, TIC of EpHCT catalyzing acyl transfer from *p*-coumaroyl CoA to shikimic acid to form *p*-coumaroyl shikimic acid. **c**, XIC of *p*-coumaroyl shikimic acid. **d**, Mass spectrum of *p*-coumaroyl shikimic acid. **e**, TIC of EpHCT catalyzing acyl transfer from caffeoyl CoA to shikimic acid to form 5-*O*-caffeoyl shikimic acid. **f**, XIC of 5-*O*-caffeoyl shikimic acid. **g**, 5-*O*-caffeoyl shikimic acid mass spectra of product and standard.

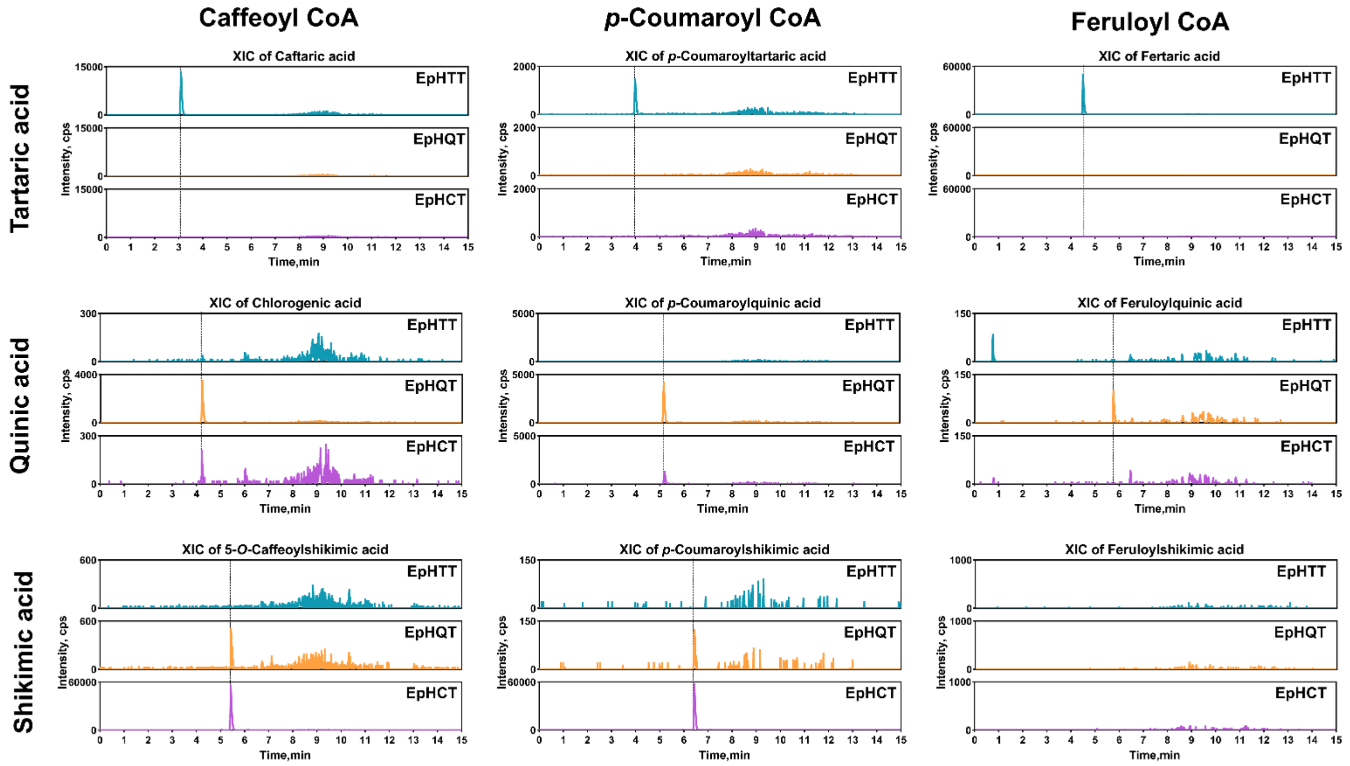

Supplementary Fig. 7. Substrate specificities of EpBAHDs. Representative XICs of different products.

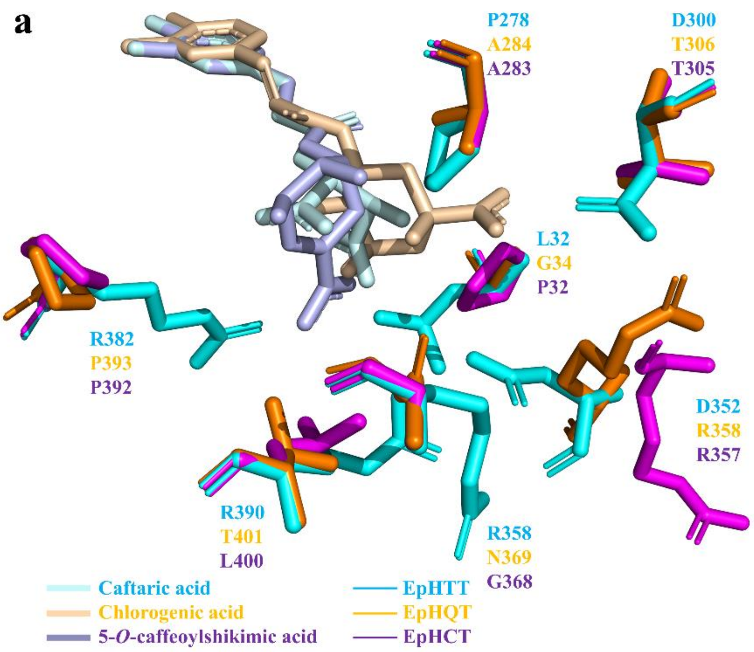

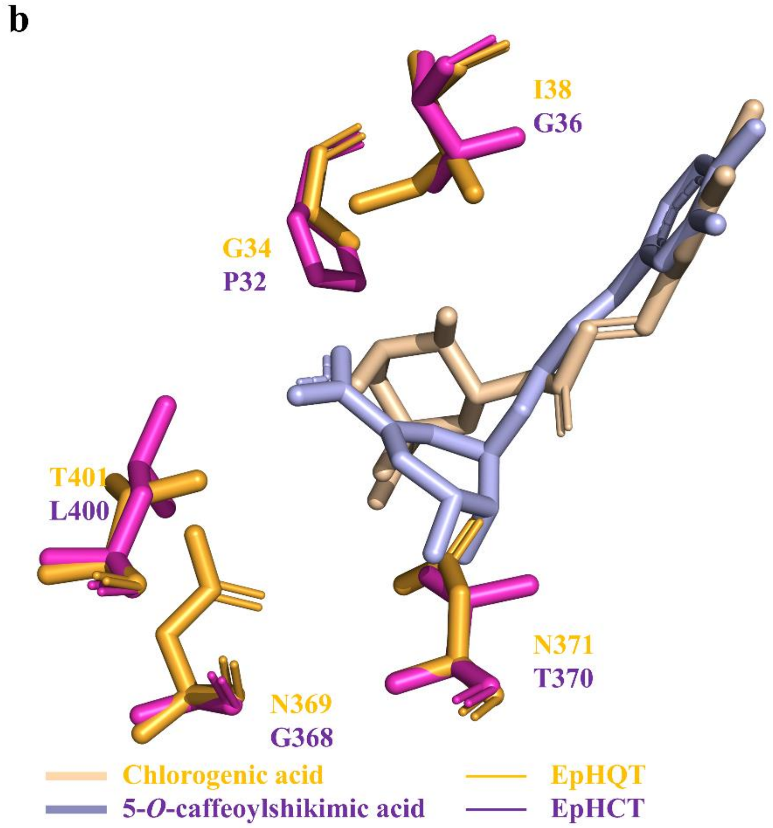

Supplementary Fig. 8. Molecular docking analysis of EpBAHDs for substrate specificities. a, Molecular docking analysis of substrate specificities of EpHTT versus EpHQT and EpHCT. **b**, Differences in the pockets between EpHQT and EpHCT.

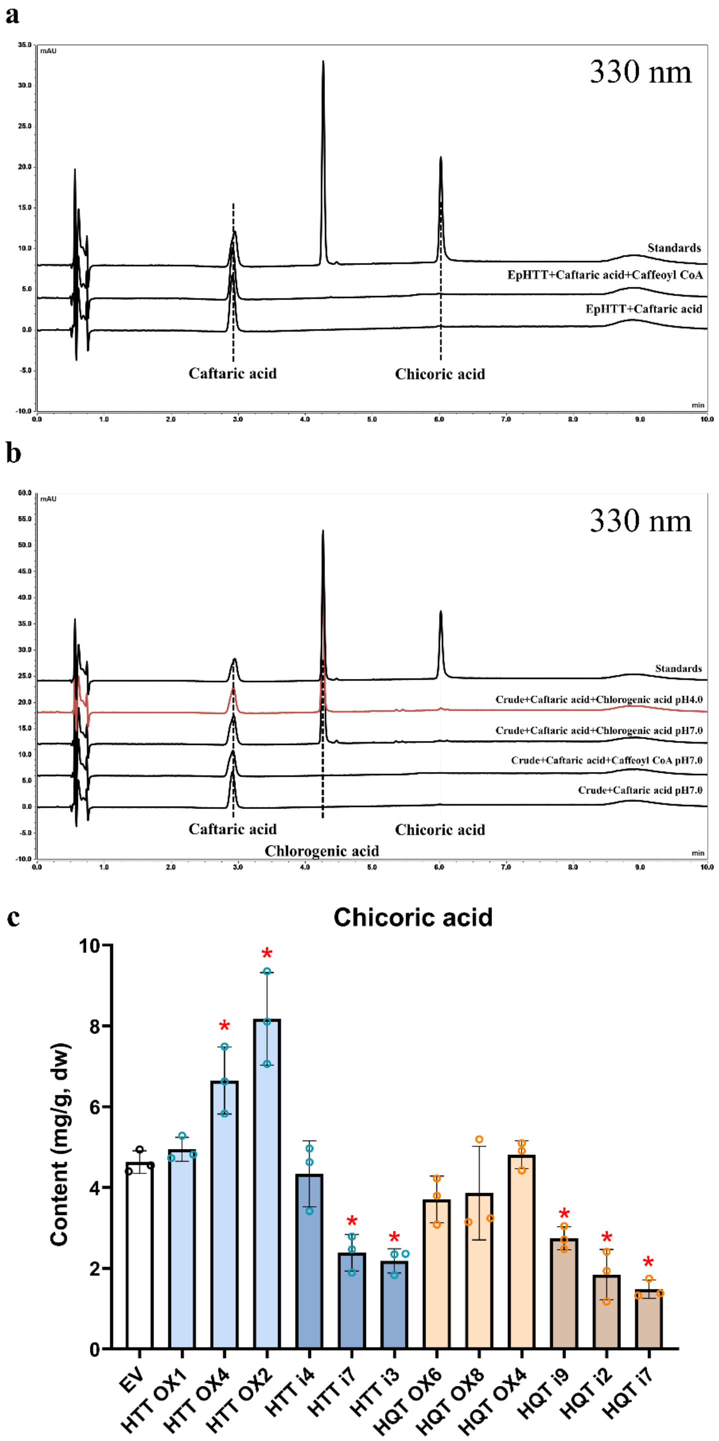

Supplementary Fig. 9. Identification of substrates for chicoric acid biosynthesis. a, UPLC chromatogram of products of incubating EpHTT with caftaric acid or caftaric acid plus caffeoyl CoA. No chicoric acid was produced. **b**, UPLC chromatogram of crude protein extract with different substrate combinations. Crude protein extracts incubated with caftaric acid plus chlorogenic acid produced chicoric acid. **c**, Chicoric acid contents in different transgenic hairy root lines. Data are mean±s.d. (n=3 biologically independent samples). * indicates significant difference from empty vector (EV) line (P<0.05) analyzed by two-sided Student's t-test. *P=0.0161 (HTT OX4), *P=0.0189 (HTT OX2), *P=0.0019 (HTT i7), *P=0.0005 (HTT i3), *P=0.0012 (HQT i9), *P=0.0021 (HQT i2), *P=0.0001 (HQT i7). Source data underlying Supplementary Figure 9c are provided as a Source Data file.

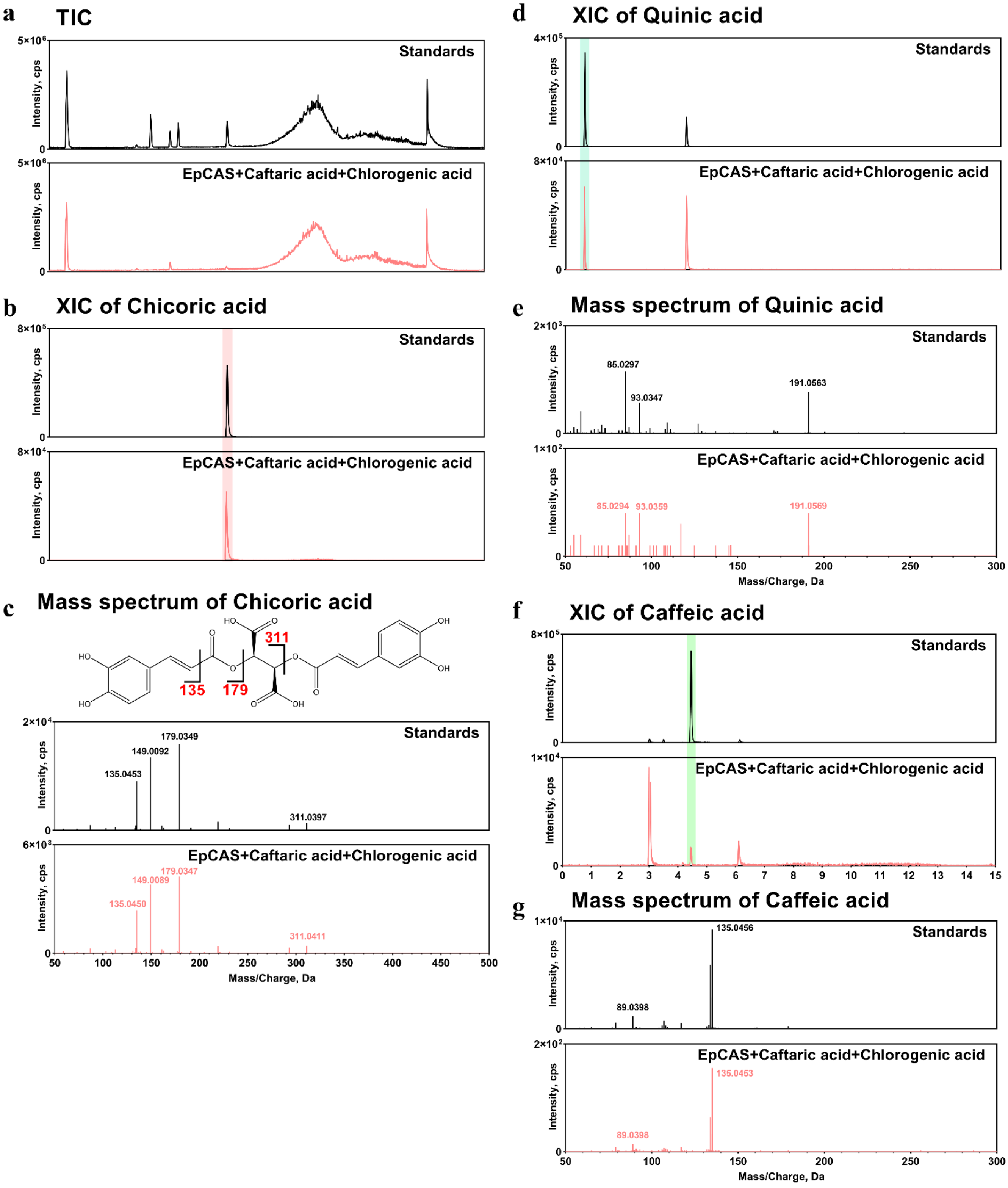

Supplementary Fig. 10. LC-HRMS analysis of the EpCAS reactions. a, TIC of standards and EpCAS reactions. **b**, XIC of chicoric acid. **c**, Chicoric acid mass spectra of product and standard. **d**, XIC of quinic acid. **e**, Quinic acid mass spectra of product and standard. **f**, XIC of caffeic acid. **g**, Caffeic acid mass spectra of product and standard.

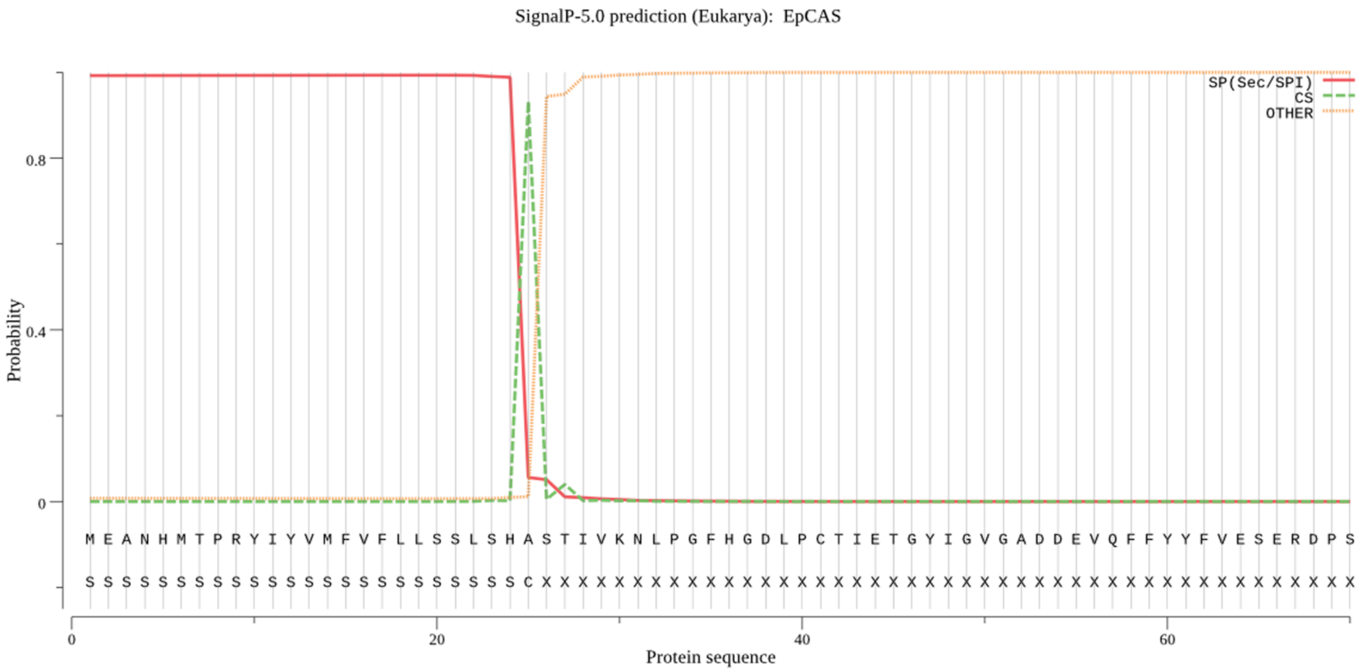

Supplementary Fig. 11. EpCAS has a signal peptide. The signal peptide was predicted by SignalP (http://www.cbs.dtu.dk/services/SignalP/). The SP (Sec/SPI) represents the likelihood probability of secretory signal peptide in EpCAS which is 0.9932. CS indicates the cleavage site between 25 and 26 amino acids. OTHER represents the probability that these amino acids in EpCAS do not have any kind of signal peptide, which is 0.0068. In the lower sequence, S indicates signal peptide amino acids; C is the cleavage site; and X indicates other amino acids.

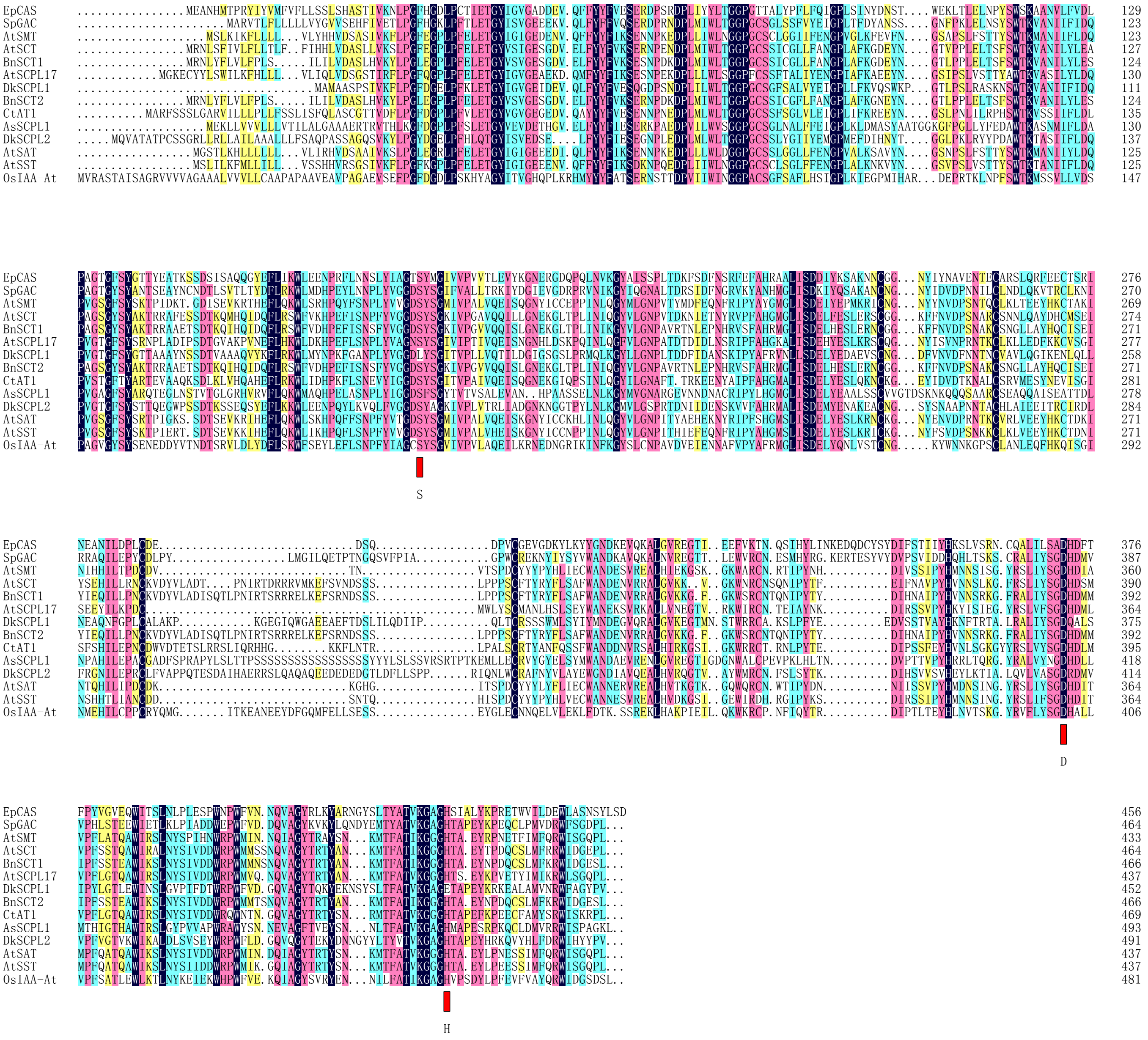

Supplementary Fig. 12. Alignment of EpCAS and other functionally characterized SCPLs. GeneBank ID of the proteins used: SpGAC, AAF64227; AtSMT, AAF78760; AtSCT, AAK52316; BnSCT1, AAQ91191; AtSCPL17, AAS99709; DkSCPL1, BAF56655; BnSCT2, CAM91991; CtAT1, BAF99695; AsSCPL1, ACT21078; DkSCPL2, BAH89272; AtSAT, AEC07395; AtSST, AEC07397; OsIAA-At, EEE56946). The conserved Ser-His-Asp catalytic triad is marked in red. The colors of dark blue, hot pink, cyan and light yellow represent 100%, ≥75%, ≥50% and ≥33% amino acid identity, respectively.

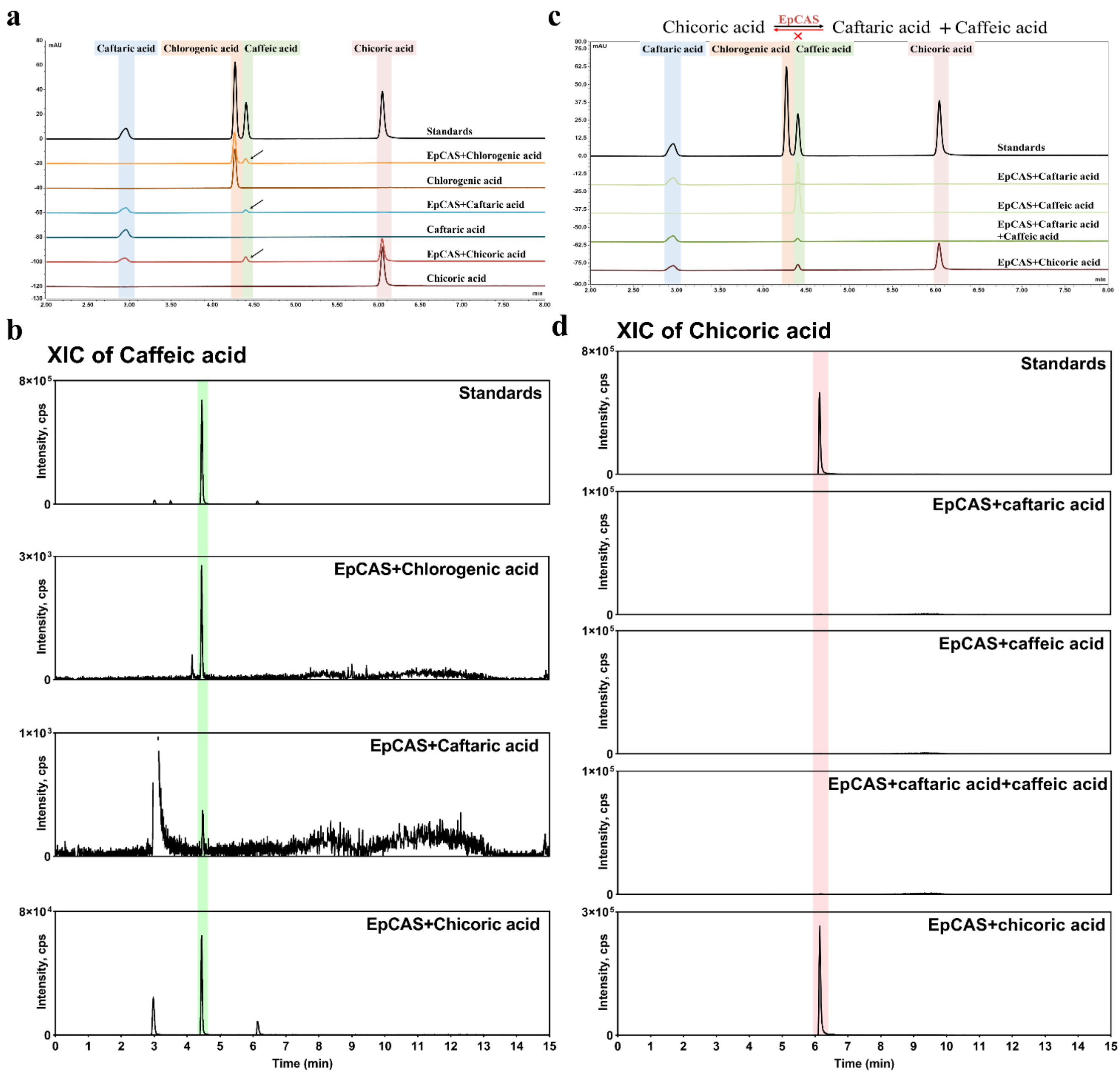

Supplementary Fig. 13. Functional diversity of EpCAS. a, UPLC chromatogram showing EpCAS can hydrolyze its substrates and product. **b**, XIC of caffeic acid measured by LC-HRMS. **c**, EpCAS cannot use caftaric acid and caffeic acid to generate chicoric acid. **d**, XIC of chicoric acid determined by LC-HRMS.

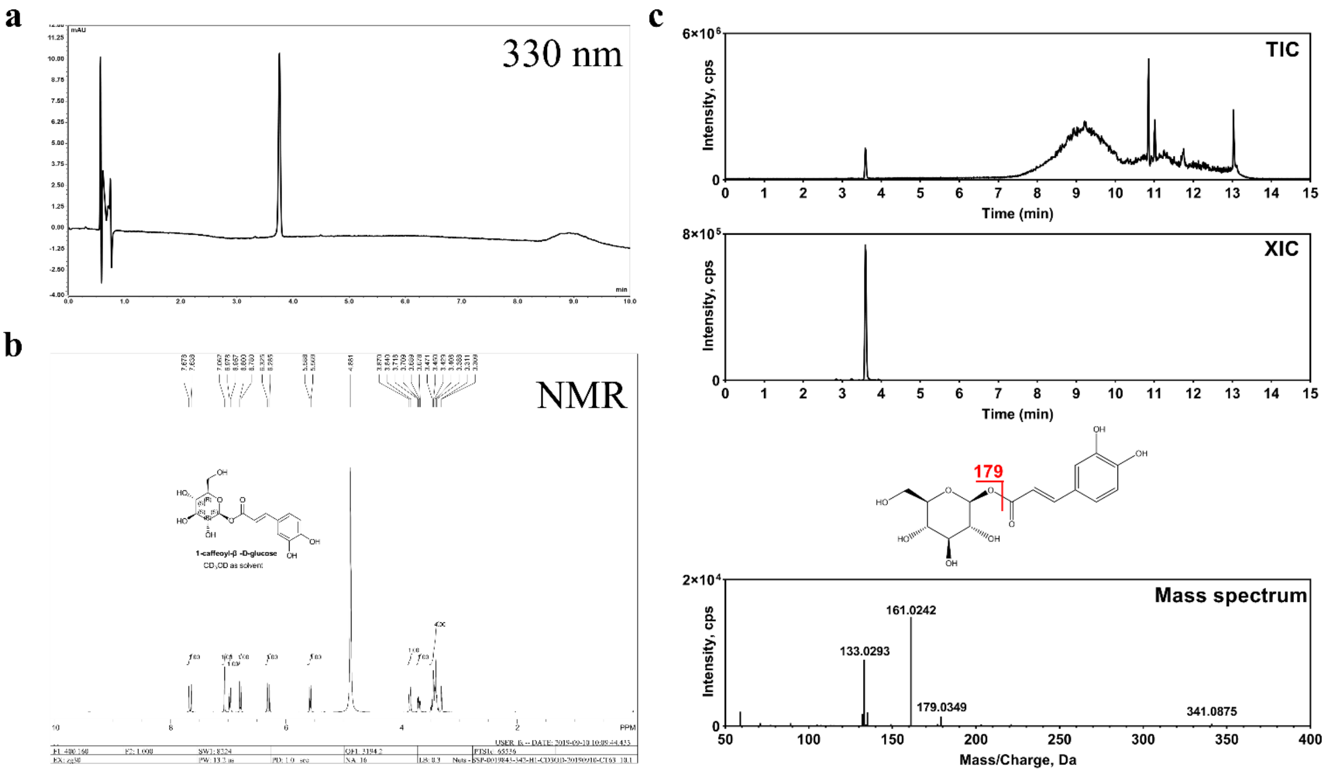

Supplementary Fig. 14. Verification of chemically synthesized 1-*O*-caffeoyl-β-D-glucose. a, UPLC-DAD analysis of 1-*O*-caffeoyl-β-D-glucose detected at 330 nm. **b**, NMR spectrum of 1-*O*-caffeoyl-β-D-glucose. **c**, LC-HRMS analysis of 1-*O*-caffeoyl-β-D-glucose.

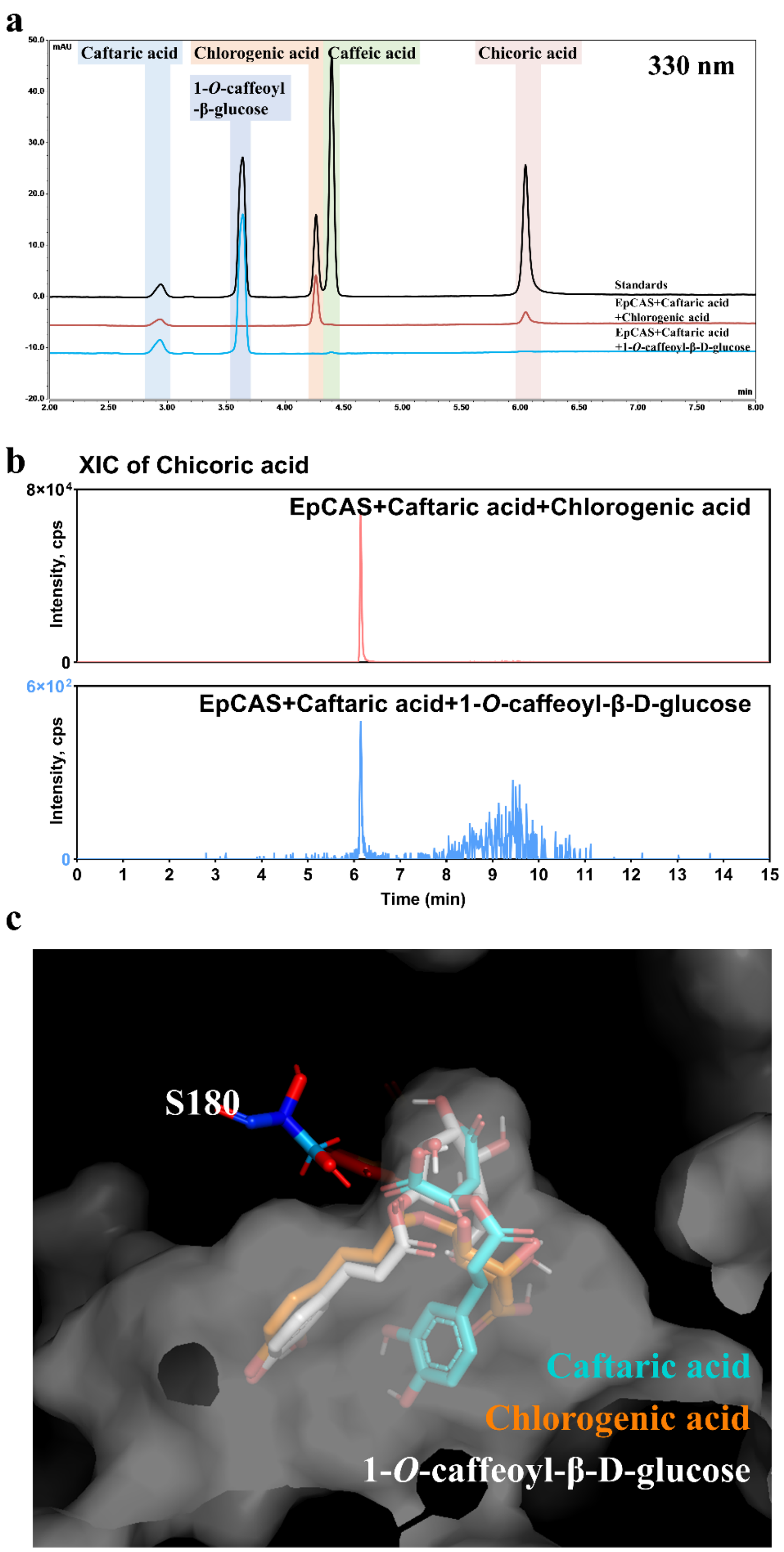

Supplementary Fig. 15. The unusual substrate preference of EpCAS. a, UPLC detection of chicoric acid generated using different acyl donors. **b,** XIC of chicoric acid detected by LC-HRMS. Chicoric acid production using 1-*O*-caffeoyl-β-D-glucose is significantly lower than that using chlorogenic acid. **c,** Molecular docking analysis of acyl donor specificity of EpCAS. Quinic acid would be liberated more easily from chlorogenic acid than glucose from 1-*O*-caffeoyl-β-D-glucose.

a

Acyl donors	Relative activity
Chlorogenic acid	100%
1-*O*-caffeoyl-β-D-glucose	0.67±0.06%*
Neochlorogenic acid	0.76±0.01%*
Cryptochlorogenic acid	0.58±0.18%*
5-*O*-caffeoyl shikimic acid	0.59±0.06%*

b

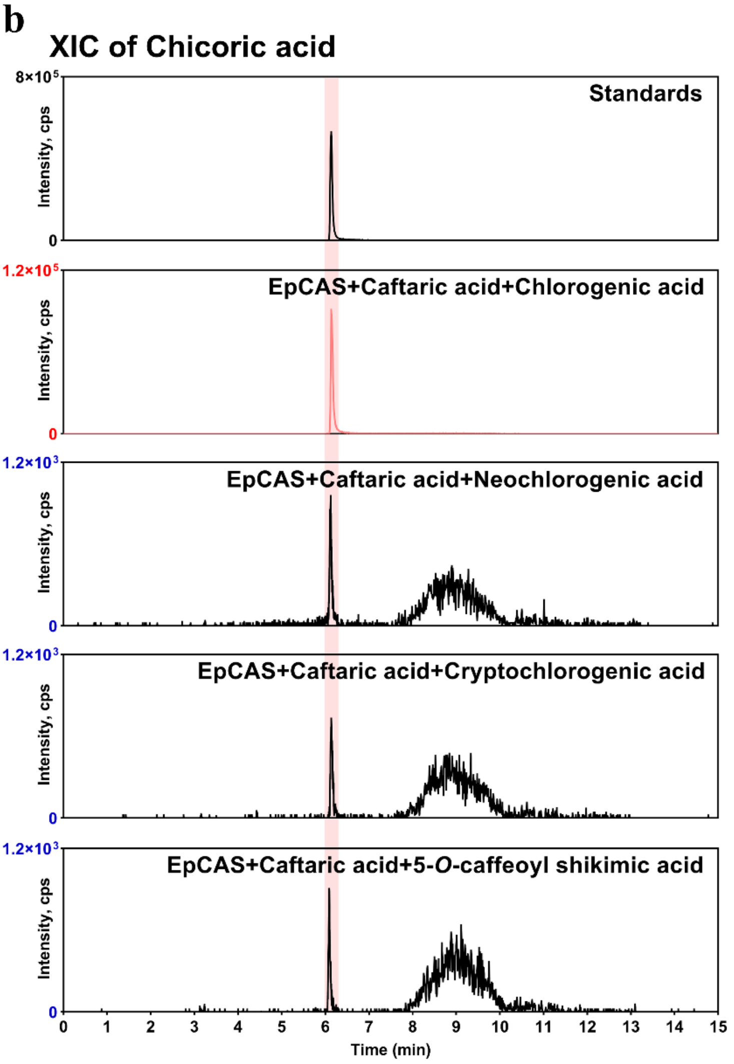

Supplementary Fig. 16. Preference of EpCAS for acyl donors. a, Relative activities of candidate acyl donors. Data are mean for chlorogenic acid and mean±s.d. for other candidate acyl donors (n=3 independent experiments). * indicates a significant difference from chlorogenic acid (P<0.05) analyzed by one-way ANOVA with Tukey's multiple comparisons test. **b**, Representative chromatograms. Source data underlying Supplementary Figure 16a are provided as a Source Data file.

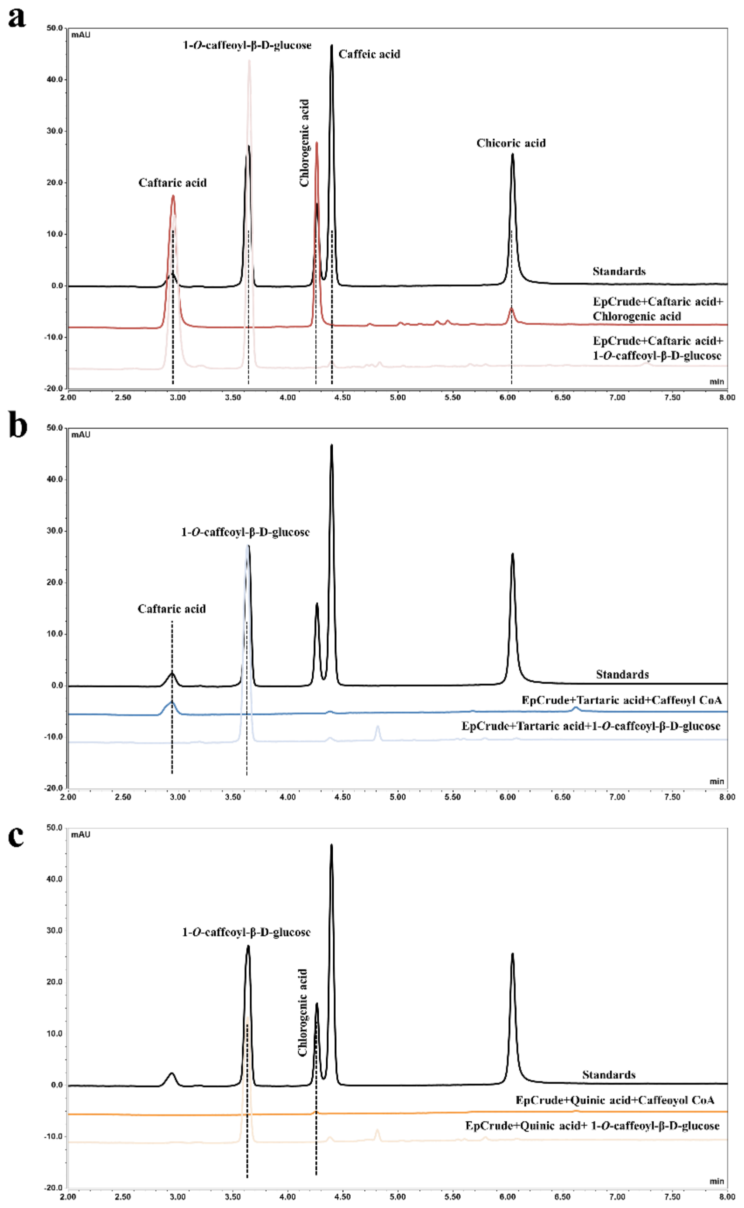

Supplementary Fig. 17. 1-*O*-caffeoyl-β-D-glucose is not an acyl donor for the biosynthesis of CADs in purple coneflower. Crude protein extract of purple coneflower could not use 1-*O*-caffeoyl-β-D-glucose as an acyl donor for the generation of **(a)** caftaric acid, **(b)** chlorogenic acid, **(c)** chicoric acid.

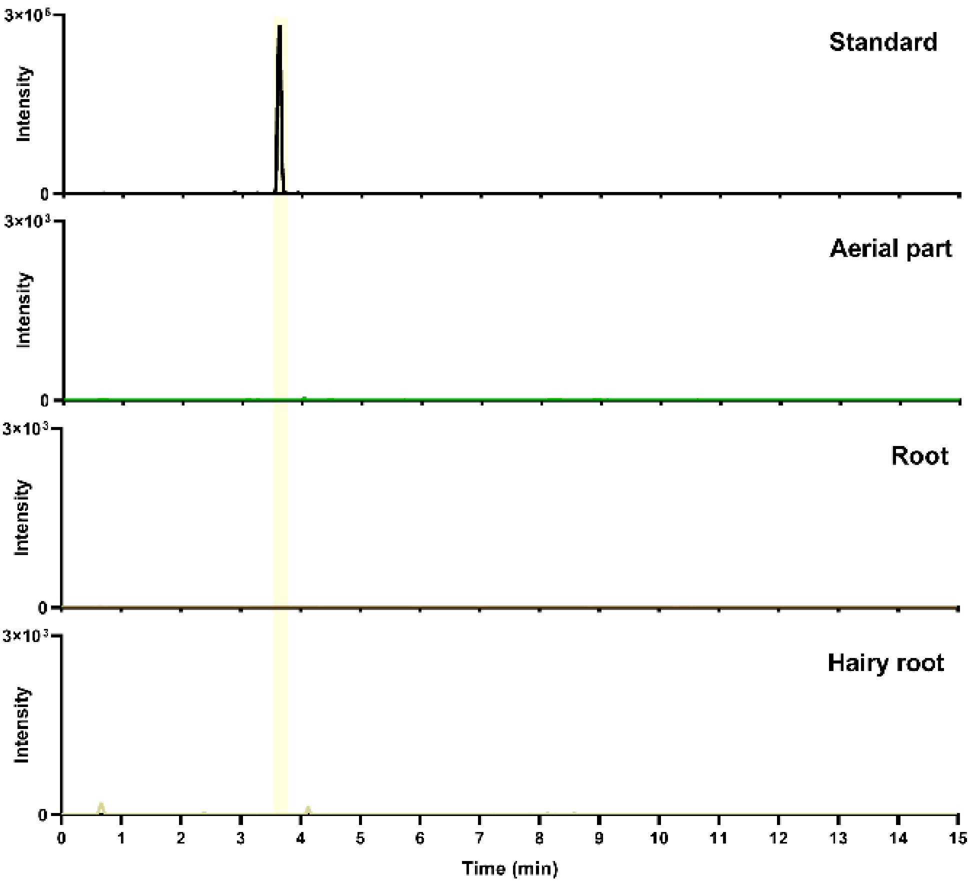

Supplementary Fig. 18. 1-*O*-caffeoyl-β-D-glucose is undetectable in purple coneflower. XIC of 1-*O*-caffeoyl-β-D-glucose from purple coneflower aerial parts, roots, and hairy root cultures.

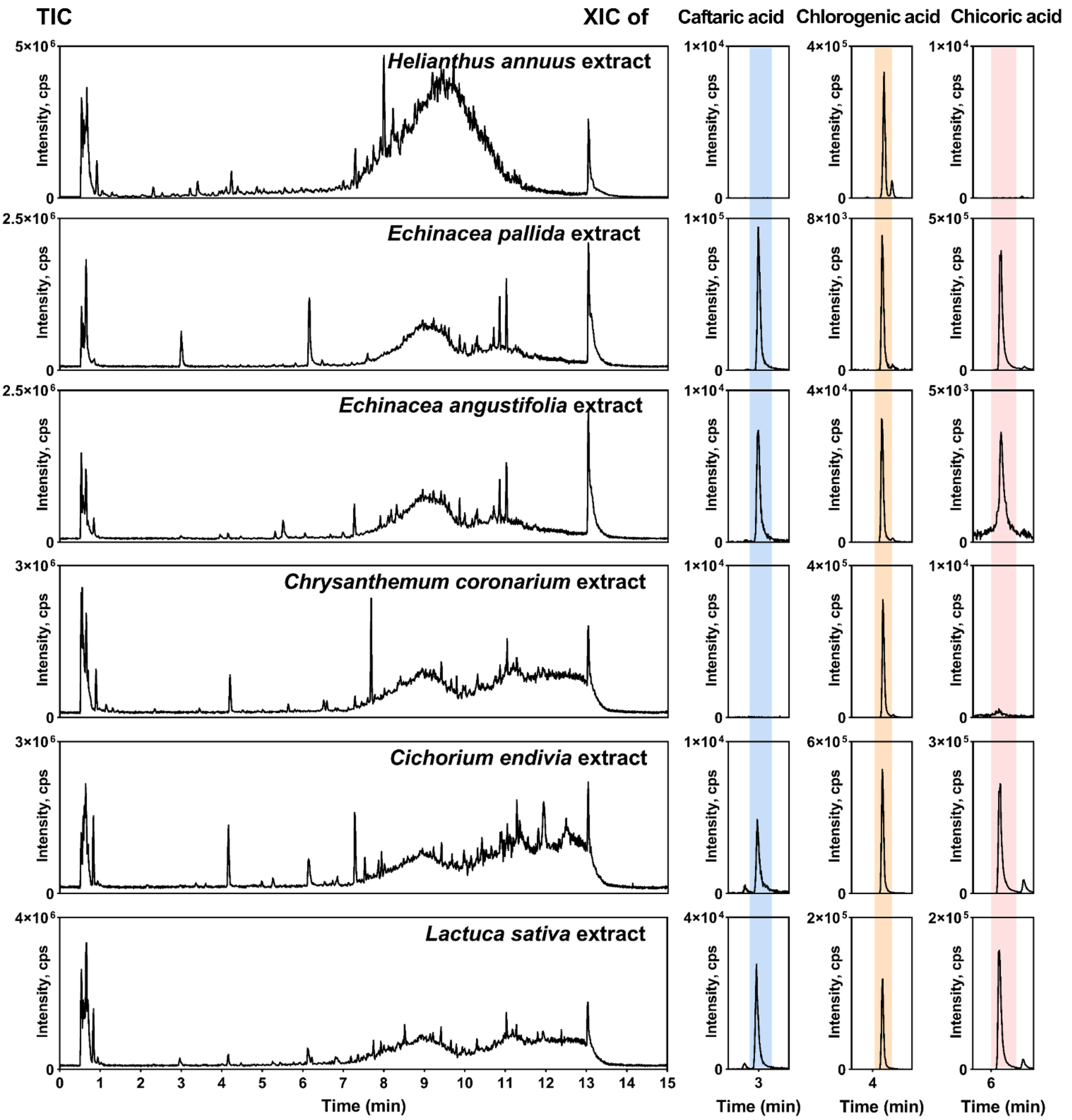

Supplementary Fig. 19. Presence of main CADs in related species. *E. pallida*, *E. angustifolia*, *C. endivia* and *L. sativa* contained all three main CADs. *H. annuus* and *C. coronarium* had only chlorogenic acid but no chicoric acid or caftaric acid. The left panels show the TIC chromatogram, and the right panels show XIC of each single compound.

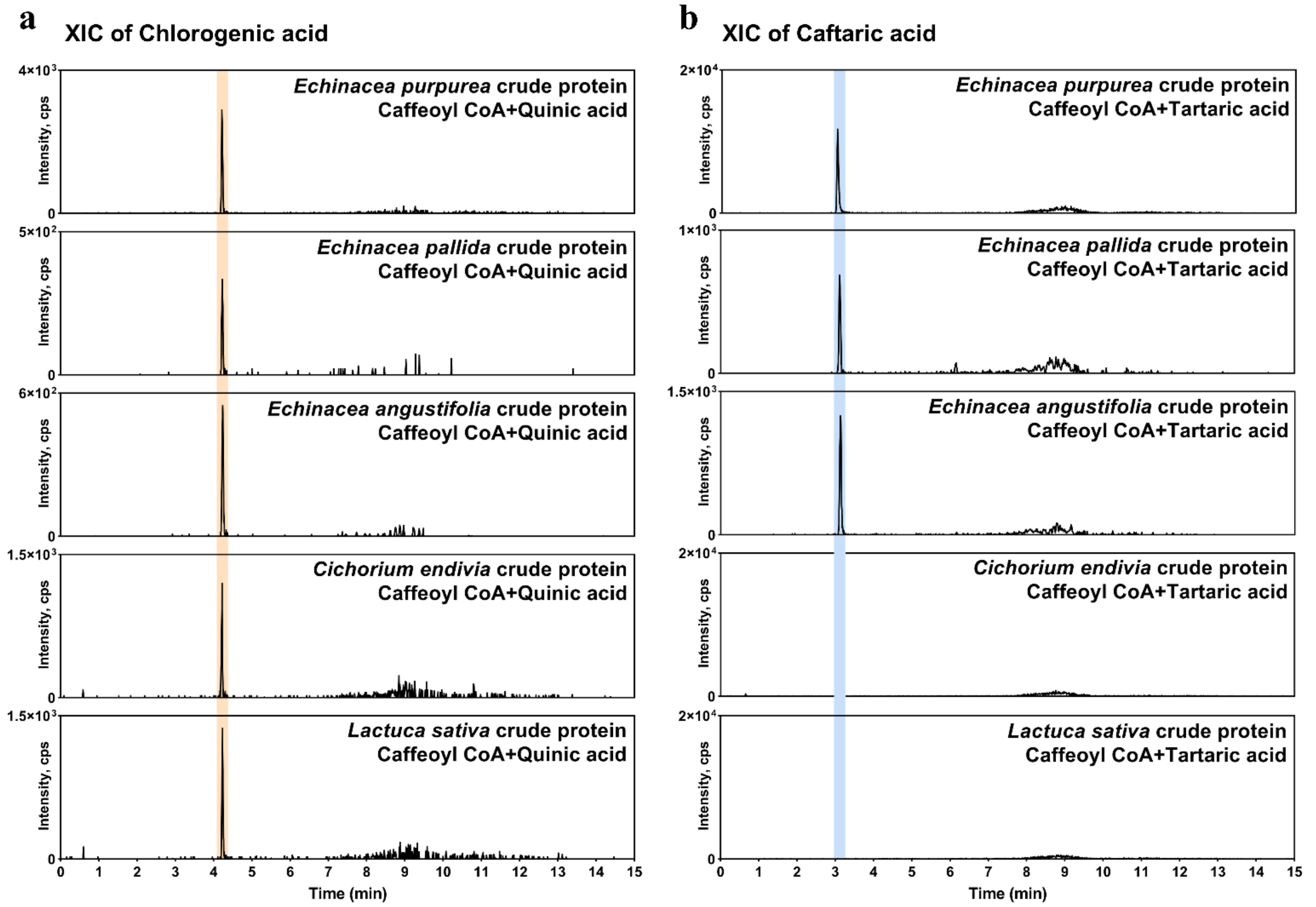

Supplementary Fig. 20. HQT and HTT activities from different plants that produce chicoric acid. a, Chlorogenic acid can be generated by incubating caffeoyl CoA and quinic acid with crude protein extracts from all species. **b**, No caftaric acid was produced by incubating caffeoyl CoA and tartaric acid with crude protein extracts from *Cichorium endivia* or *Lactuca sativa*, although it was produced by crude extracts of all three *Echinacea* species.

Supplementary Table 1. The kinetic characterizes of EpHTT.

Substrates			EpHTT		
Fixed	Concentration	Change	*Km* (μM)	*Kcat* (s^{-1})	*Kcat*/*Km* (s^{-1} mM^{-1})
Tartaric acid	0.5 mM	Caffeoyl CoA	34.760±6.832	0.588±0.064	17.127±1.585 (100%)
Tartaric acid	0.5 mM	*p*-Coumaroyl CoA	22.027±3.818	0.007±0.001	0.343±0.054 (2%)
Tartaric acid	0.5 mM	Feruloyl CoA	48.010±11.329	0.122±0.011	2.697±0.975 (15.7%)
Caffeoyl CoA	50 μM	Tartaric acid	841.967±35.474	0.685±0.016	0.814±0.025
p-Coumaroyl CoA	100 μM	Tartaric acid	2182.667±7944.749	0.017±0.004	0.008±0.002
Feruloyl CoA	100 μM	Tartaric acid	458.600±80.431	0.176±0.052	0.390±0.122

Data are expressed as mean±s.d. with three independent experiments. The quantification of *p*-Coumaroyltartaric acid and feruloyltartaric acid were based on caftaric acid as reference. Source data are provided as a Source Data file.

Supplementary Table 2. The kinetic characterizes of EpHQT.

Substrates			EpHQT		
Fixed	Concentration	Change	*Km* (μM)	*Kcat* (s^{-1})	*Kcat*/*Km* (s^{-1} mM^{-1})
Quinic acid	1 mM	Caffeoyl CoA	7.231±1.577	0.111±0.005	15.752±2.893 (100%)
Quinic acid	1 mM	*p*-Coumaroyl CoA	70.547±43.952	0.060±0.036	0.992±0.669 (6.3%)
Quinic acid	1 mM	Feruloyl CoA	38.623±14.588	0.0007±0.00009	0.019±0.007 (0.1%)
Caffeoyl CoA	50 μM	Quinic acid	576.167±72.176	0.157±0.007	0.274±0.023
p-Coumaroyl CoA	100 μM	Quinic acid	361.225±105.511	0.085±0.044	0.212±0.125
Feruloyl CoA	100 μM	Quinic acid	6002.333±744.151	0.003±0.001	0.0006±0.00001

Data are expressed as mean±s.d. with three independent experiments. Chlorogenic acid was used as reference for the measurement of *p*-Coumarylquinic acid and feruloylquinic acid. Source data are provided as a Source Data file.

Supplementary Table 3. The kinetic characterizes of EpCAS.

Fixed	Chlorogenic acid 200 μM	Caftaric acid 200 μM	Caftaric acid 200 μM
Changed	Caftaric acid	Chlorogenic acid	1-*O*-caffeoyl-β-D-glucose
Km (μM)	53.910±0.613	83.910±7.811	121.400±12.163
Kcat (s^{-1})	0.005±0.00002	0.005±0.0003	1.090×10^{-5}±0. 087×10^{-5}
Kcat/Km (s^{-1} mM^{-1})	0.086±0.0007	0.062±0.003	8.997×10^{-5}±0.247×10^{-5}

Data are expressed as mean±s.d. with three independent experiments. Source data are provided as a Source Data file.

Supplementary Table 4. Nucleotide sequences of the primers used in this study.

Primer	Nucleotide sequence
For clone of target gene into pDONR207 vector for Gateway cloning	
Underlined sequences mean recombination cites for Gateway cloning	
EpHTT_F	GGGGACAAGTTTGTACAAAAAAGCAGGCTTAATGAAGGTGGTGGTTAGAGAATCCAC
EpHTT_R	GGGGACCACTTTGTACAAGAAAGCTGGGTATCAGATTGCATACAACAAGTTGCC
EpHQT_F	GGGGACAAGTTTGTACAAAAAAGCAGGCTTAATGAACATAACAATAACCAAATCATC
EpHQT_R	GGGGACCACTTTGTACAAGAAAGCTGGGTATTAAAACTCATACAAATACTTCTCA
EpCAS_F	GGGGACAAGTTTGTACAAAAAAGCAGGCTTAATGGAGGCTAATCACATGACGCCA
EpCAS_R	GGGGACCACTTTGTACAAGAAAGCTGGGTATTAAGAGTCGCTCAAATAAGAATT
EpHTTi_F	GGGGACAAGTTTGTACAAAAAAGCAGGCTTACGATGTGGAAACAAAGCTCTATCTC
EpHTTi_R	GGGGACCACTTTGTACAAGAAAGCTGGGTAAAGGCTTTTTACAATTGTGTTGTTC
EpHQTi_F	GGGGACAAGTTTGTACAAAAAAGCAGGCTTACCTGTTTTTATGGGACCCGCGATC
EpHQTi_R	GGGGACCACTTTGTACAAGAAAGCTGGGTATTAAAACTCATACAAATACTTCTCA
EpCASi_F	GGGGACAAGTTTGTACAAAAAAGCAGGCTTAAAAGTCCATGGAACCCATG
EpCASi_R	GGGGACCACTTTGTACAAGAAAGCTGGGTAGAGTCGCTCAAATAAGAAT
For expression of recombinant proteins in yeast using pESC-His vector	
EpCAS_F	CTATAGGGCCCGGGCGTCGACATGGAGGCTAATCACATGACGCCA
EpCAS_R	GCGGTACCAAGCTTACTCGAGTTAATGATGGTGATGGTGATGAGAGTCGCTCAAATAAG
EpCAS-S180A_F	GCTGGGACTGCATACATGGGTATAG
EpCAS-S180A_R	CTATACCCATGTATGCAGTCCCAGC
EpCAS-D374A_F	GCTGATCATGATGCAACGTTTCCATATG
EpCAS-D374A_R	CATATGGAAACGTTGCATCATGATCAGC
EpCAS-H430A_F	GCTGGCCATGCAATCGCGTTATAC
EpCAS-H430A_R	GTATAACGCGATTGCATGGCCAGC
For subcellular localization using pSuper1300-GFP vector	

EpHTT_F	CGCGTCGACATGAAGGTGGTGGTTAGAGAATCCAC
EpHTT_R	GCGGTACCGATTGCATACAACAAGTTGCC
EpHQT_F	CGCGTCGACATGAACATAACAATAACCAAATCATC
EpHQT_R	GCGGTACCAAACTCATACAAATACTTCTC
EpCAS_F	CGCGTCGACATGGAGGCTAATCACATGACGCCA
EpCAS_R	GCGGTACCAGAGTCGCTCAAATAA

For qPCR

EpHTT_F	TTAAGTCGAAACCTATCTGGTATGC
EpHTT_R	AAGGCTTTTTACAATTGTGTTGTTC
EpHQT_F	TGGGACCCGCGATCATATTG
EpHQT_R	GCATACTGCCAAAGACACACT
EpCAS_F	CAACTGTTAAGGGTGCTGGC
EpCAS_R	GCCATTCATCCAAAATGACCCA
EpTubulin_F	TGAAGACATTGACGGGGAAG
EpTubulin_R	CACCACGAAGACGAAGAAC
