# Supplementary material for: Versatility in acyltransferase activity completes chicoric acid biosynthesis in purple coneflower
Source: Nat Commun. 2021 Mar 10;12:1563. doi: 10.1038/s41467-021-21853-6 (PMC7946891; doi:10.1038/s41467-021-21853-6)
Supplement: Supplementary file 3 — Reporting Summary [file 41467_2021_21853_MOESM3_ESM.pdf]

Corresponding author(s): Yang Zhang

Last updated by author(s): Feb 6, 2021

Reporting Summary

Nature Research wishes to improve the reproducibility of the work that we publish. This form provides structure for consistency and transparency in reporting. For further information on Nature Research policies, see our Editorial Policies and the Editorial Policy Checklist.

Statistics

For all statistical analyses, confirm that the following items are present in the figure legend, table legend, main text, or Methods section.

n/a	Confirmed	
☐	☒	The exact sample size (*n*) for each experimental group/condition, given as a discrete number and unit of measurement
☐	☒	A statement on whether measurements were taken from distinct samples or whether the same sample was measured repeatedly
☐	☒	The statistical test(s) used AND whether they are one- or two-sided<br>*Only common tests should be described solely by name; describe more complex techniques in the Methods section.*
☐	☒	A description of all covariates tested
☐	☒	A description of any assumptions or corrections, such as tests of normality and adjustment for multiple comparisons
☐	☒	A full description of the statistical parameters including central tendency (e.g. means) or other basic estimates (e.g. regression coefficient) AND variation (e.g. standard deviation) or associated estimates of uncertainty (e.g. confidence intervals)
☐	☒	For null hypothesis testing, the test statistic (e.g. F, t, r) with confidence intervals, effect sizes, degrees of freedom and P value noted<br>*Give P values as exact values whenever suitable.*
☒	☐	For Bayesian analysis, information on the choice of priors and Markov chain Monte Carlo settings
☒	☐	For hierarchical and complex designs, identification of the appropriate level for tests and full reporting of outcomes
☒	☐	Estimates of effect sizes (e.g. Cohen's *d*, Pearson's *r*), indicating how they were calculated

Our web collection on statistics for biologists contains articles on many of the points above.

Software and code

Policy information about availability of computer code

Data collection	SCIEX OS (version 1.7); Chromeleon (version 7.2 SR4); Analyst TF (version 1.7).
Data analysis	ProteinPilot (version 5.0.2); I-TASSER (https://zhanglab.ccmb.med.umich.edu/I-TASSER); Swiss-Model (https://swissmodel.expasy.org/); CIS-RR (http://jianglab.ibp.ac.cn/lims/cisrr/cisrr.html); CB-Dock (http://clab.labshare.cn/cb-dock/php/); MEGA6 (version 6.0); AutoDock (version 4.2); PyMOL (version 2.3.2); GraphPad (version 8.02); Microsoft Excel (version Office 365 2019); Bio-Rad CFX manager (version 3.1).

For manuscripts utilizing custom algorithms or software that are central to the research but not yet described in published literature, software must be made available to editors and reviewers. We strongly encourage code deposition in a community repository (e.g. GitHub). See the Nature Research guidelines for submitting code & software for further information.

Data

Policy information about availability of data

All manuscripts must include a data availability statement. This statement should provide the following information, where applicable:

- Accession codes, unique identifiers, or web links for publicly available datasets
- A list of figures that have associated raw data
- A description of any restrictions on data availability

The sequences of the genes reported in this article have been deposited in NCBI GenBank (accessions MT936803, MT936804, MT936805, MT936806). The RNA-seq data from MeJA treatment and the control have been deposited in the SRA database under accession numbers SRR8935731, SRR8935732, SRR8935733 (MeJA) and SRR8937034, SRR8937035, SRR8937036 (Control). Source data are provided with this paper. Additional data that support the findings of this study are available from the corresponding authors upon request.

Field-specific reporting

Please select the one below that is the best fit for your research. If you are not sure, read the appropriate sections before making your selection.

☒ Life sciences ☐ Behavioural & social sciences ☐ Ecological, evolutionary & environmental sciences

For a reference copy of the document with all sections, see nature.com/documents/nr-reporting-summary-flat.pdf

Life sciences study design

All studies must disclose on these points even when the disclosure is negative.

Sample size	A minimum sample size of 3 independent biological experiments was chosen to obtain valid statistical analyses. Sample size has been described in each individual experiment.
Data exclusions	No data were excluded from the analyses.
Replication	All replication were successful performed independently. Number of replication has been describe in each individual experiment/figure legend
Randomization	The LC-HRMS and UPLC analysis orders were randomized.
Blinding	No investigations were blinded because blinding was not seriously relevant to our experiments (non-clinical trials).

Reporting for specific materials, systems and methods

We require information from authors about some types of materials, experimental systems and methods used in many studies. Here, indicate whether each material, system or method listed is relevant to your study. If you are not sure if a list item applies to your research, read the appropriate section before selecting a response.

Materials & experimental systems

n/a	Involved in the study
☒	☐ Antibodies
☒	☐ Eukaryotic cell lines
☒	☐ Palaeontology and archaeology
☒	☐ Animals and other organisms
☒	☐ Human research participants
☒	☐ Clinical data
☒	☐ Dual use research of concern

Methods

n/a	Involved in the study
☒	☐ ChIP-seq
☒	☐ Flow cytometry
☒	☐ MRI-based neuroimaging
